# The effect of resistance training on health-related quality of life in older adults: Systematic review and meta-analysis

**DOI:** 10.15171/hpp.2019.01

**Published:** 2019-01-23

**Authors:** Peter D. Hart, Diona J. Buck

**Affiliations:** ^1^Health Promotion Program, Montana State University - Northern, Havre, MT 59501, USA; ^2^Kinesmetrics Lab, Montana State University - Northern, Havre, MT 59501, USA

**Keywords:** Systematic review, Meta-analysis, Resistance training, Health-related quality of life, SF-36

## Abstract

**Background:** Resistance training (RT) is recommended as part of our national physical activity guidelines which includes working all major muscle groups on two or more days a week.Older adults can gain many health benefits from RT, such as increased muscle strength,increased muscle mass, and maintenance of bone density. Additionally, certain dimensions of health-related quality of life (HRQOL) have been shown to improve in older adults due to RT intervention. The purpose of this study was to use systematic review and meta-analytic techniques to examine the effect of RT on HRQOL in older adults.

**Methods:** A systematic review of current studies (2008 thru 2017) was conducted using PubMed. Studies were included if they used a randomized controlled design, had RT as an intervention, measured HRQOL using the SF-36/12 assessment, and included adults 50+ years of age. Eight dimension scores (physical functioning, bodily pain, physical role function, general health, mental health, emotional role function, social function, and vitality) and two summary scores (physical component and mental component) were extracted. Ten meta-analyses were performed using standardized mean effect sizes and random effects models. Study quality,moderator and sensitivity analyses were conducted.

**Results: ** A total of 16 studies were included in the analyses with a mean Physiotherapy Evidence Database (PEDro) score of 4.9 (SD=1.0). Among the mental health measures, RT had the greatest effect on mental health (Effect size [ES]=0.64, 95% CI: 0.30-0.99, I^2^=79.7). Among the physical health measures, RT had the largest effect on body pain (ES=0.81, 95% CI: 0.26-1.35, I^2^=85.9).Initially, RT did not significantly affect measures of emotional role function, social function or physical role function. However, after removing a single study, RT significantly increased all HRQOL measures.

**Conclusion:** The meta-analytic evidence presented in this research clearly supports the promotion of RT in improving HRQOL in older adults.

## Introduction

Resistance training (RT) is recommended as part of the 2008 physical activity guidelines for Americans.^1^ Specifically, adults should engage in muscle strengthening activities of moderate to high intensity which includes working all major muscle groups on two or more days a week. For the aged adult, the same muscle strengthening guidelines apply, as RT may hold even greater benefit for this population. Several health problems affecting older adults can be countered by adopting a regular RT program. For example, older adults are at greater risk of premature death due to falls, which is associated with age-related declines in muscular fitness and balance.^2-5^ A recent report from the Centers for Disease Control and Prevention (CDC) states that approximately one in four older (65+ years of age) US adults fall each year and deaths from falls have increased an average of 3% annually from 2007 to 2016.^6^

Older adults can gain other health benefits from RT, besides increased muscle mass and strength.^7^ Studies have shown that RT can benefit bone mineral density,^8,9^ lipoprotein profiles,^10^ glycemic control,^11^ body composition,^12^ symptoms of frailty,^13^ metabolic syndrome risk factors,^14^ and cardiovascular disease markers.^15^ Studies have further shown that RT can decrease the risk of all-cause mortality both in observational^16,17^ and experimental^18^ designs. Furthermore, RT intervention has shown to effectively improve psychosocial health outcomes such as sense of coherence,^19^ perceived stress,^20^ depression,^21^ anxiety,^22^ and fatigue.^23^

Health-related quality of life (HRQOL) is another psychosocial outcome of increasing interest in health sciences research.^24^ HRQOL is a multidimensional construct and considers the relationship between an individual’s health status and their quality of life.^25^ As a recent addition to the *Healthy People* goals for year 2020, two objectives were issued.^26^ Specifically, these objectives are to increase the proportion of adults who report at least good health, with one objective specifying physical health and the other specifying mental health.

As with any health outcome measure used in practice or in research, the use of a reliable HRQOL measure is important to the internal validity of study findings.^27^ Therefore, selecting an appropriate assessment is paramount to research soundness. Many different HRQOL assessments have been used in physical activity-related research, however, the Medical Outcomes Study 36-Item Short Form Survey (SF-36) and its variant (SF-12) have served as a gold-standard.^28^ One attractive characteristic of the SF-36 and SF-12 assessments (SF-36/12), is the many different outcome scores resulting from its administration. Specifically, ten different scores can be computed from the SF-36/12: eight dimension scores (physical functioning [PF], bodily pain [BP], physical role function [PRF], general health [GH], mental health [MH], emotional role function [ERF], social function [SF], and vitality [VT]) and two summary scores (physical component [PCS] and mental component [MCS]).^29^

Due to its widespread use and above standard psychometric properties,^30^ this research delimited its examination to only studies using the SF-36/12 to measure HRQOL. Moreover, studies support the positive effect that RT has on HRQOL.^31^ However, a collective summary of the effect that RT has on a gold-standard HRQOL assessment is necessary. A collective summary through systematic review can ensure that the promotion and adoption of RT among older adults will contribute to the effort to meet our national HRQOL objectives. Therefore, the purpose of this study was to use systematic review and meta-analytic techniques to examine the effect of RT on HRQOL, assessed only using the SF-36/12, among older adults.

## Material and Methods

### Systematic review search strategy

Two researchers independently engaged in all search strategy procedures. During review of the results at each stage, if discrepancies were found between the researchers, they were reviewed and discussed until an agreement was made. The search strategy steps consisted of: (1) initial search of the PubMed database using keyword search terms and review of all initial abstracts, (2) retrieval and review of all full-text articles estimated to be appropriate from the initial abstract review, and (3) agreement on the final set of full-text articles included in the study. The following terms were used in the initial PUBMED search: “(elderly OR older OR aging) (“strength training” OR “resistance training” OR “resistance exercise” OR “muscle strengthening” OR “weight training”) (“HRQOL” OR “SF-36” OR “SF-12” OR “health-related quality of life” OR PCS OR MCS OR “physical component” OR “mental component”)”.

### Inclusion and exclusion criteria

During the search procedures stated above, inclusion criteria were used to flag abstracts and full-text articles as appropriate for the study. During the last stage of the search, included studies were excluded only if the data reported were not conducive to a standardized mean difference meta-analysis (e.g., regression analysis). The following inclusion criteria were used during each step of the search:

1. *Date criteria*: The full-text articles must have been published within a 10-year period, beginning January 2008 and ending December 2017.
2. *Article criteria*: The full-text articles had to represent first-hand research.
3. *Study design criteria*: The full-text articles must have examined research outcomes from a randomized controlled pretest-posttest design. Single group studies and studies with between-group posttest only results were not included.
4. *Age criteria*: The full-text articles must have examined the effect of RT of HRQOL using adults at least 50 years of age. If studies included younger and older subjects but did not stratify the analyses by age groups to focus on the 50+ age group, they were not included.
5. *Intervention criteria:* The full-text articles must have examined research that used RT as a primary treatment/exposure/intervention component. RT was defined as any regimented program that worked large muscle groups by using either concentric, eccentric, or isometric muscle actions.
6. *HRQOL criteria:* The research studies must have measured HRQOL using the SF-36/12 assessment.
7. *Posttest criteria*: The research studies had to have measured HRQOL after the RT program. Studies that only examined long-term effects of RT on HRQOL were not included.

### Data extraction

Data were extracted from each study independently by the same two researchers. A preformatted spreadsheet was created for both researchers and included the following columns: study number, first author last name, year of publication, HRQOL form (SF-36 or SF-12), mean age of participants, minimum age of participants, gender of participants (male/female/both), disease status (e.g., diabetic) (yes or no), length of intervention (in weeks), and whether the intervention included other components (i.e., multiplicity) (RT only or RT plus). Additionally, data for effect size calculations were extracted and included any number of columns such as pretest mean value and standard deviation (SD), posttest mean value and SD, mean gain score value and SD, between group mean difference in gains and SD, confidence interval limits, standard errors (SEs), *P* values, and test statistics. All data were entered into spreadsheets with the same formatting and a comparison of results was performed using the SAS PROC COMPARE procedure.^32^ Any discrepancies in data extraction were discussed until an agreement was made.

### Statistical analysis

Each of the ten HRQOL measures from the SF-36/12 assessment was considered distinct measures of HRQOL and so ten different meta-analyses were performed. Each meta-analysis was conducted using the computed standardized mean effect size (ES) and its SE.^33^ The effect size in this study represents the effect that RT has on HRQOL, as compared to a control. In 15 of the 16 studies, the reported pretest and posttest means and standard deviations were used to compute each effect size. The numerator of each effect size was simply the difference between the treatment mean difference and the control mean difference. The denominator of each effect size was a pooled standard deviation of the two group’s standard deviation of changes. When these standard deviations of changes were not reported, we estimated them using conventional methods.^34^ When pretest and posttest group standard deviations were not reported directly, we computed them from reported confidence intervals. The effect size standard errors were also calculated using the computed effect size and group sample sizes. The one unique study reported all change statistics for each group. In this case, the effect size numerator was simply the subtraction between the two reported mean differences. In this case, the denominator was the conventional pooled standard deviation of the two reported standard deviations of changes.

For this meta-analysis research, it was assumed that different populations indeed exist within the older adult population (e.g., diseased and non-diseased) and therefore RT would have varying effect on HRQOL across these different populations. With this assumption in mind, random effects models were pre-planned and performed on all meta-analyses.^35^ To describe individual study-level effect sizes and each pooled effect size, Forest plots were constructed with 95% confidence intervals (CIs).^36^ To further describe variability in effect sizes, the *Q* statistic for heterogeneity, tau-squared (*τ*^2^) representing the variance component, and *I*^2^ describing percent of heterogeneity were computed.^37^ Additionally, moderator analyses using random effects models were performed for four categorical factors and three continuous factors.^35^

Three procedures were employed as part of a sensitivity analysis. First, Egger’s regression was performed to test funnel plot asymmetry.^38^ Second, a trim-and-fill procedure was performed to estimate the number of effect sizes needed to reproduce a symmetric funnel plot.^39^ An estimated mean effect size was produced as part of the trim-and-fill analysis and represents the change in pooled effect size with imputed study effect sizes required to balance each funnel plot. Third, a leave-one-out analysis was performed which estimates new pooled effect size estimates with each study deleted.^40^ Finally, the experimental design quality of each study included in this research was evaluated using the Physiotherapy Evidence Database (PEDro) scale.^41^ SAS version 9.4 (SAS Institute, Cary, NC, USA),^32^ R version 3.5 (R Core Team, Vienna, Austria),^42^ and STATA version 14 (StataCorp, College Station, TX, USA) ^43^ were used for all analyses. Significance was set to *P *< 0.05. Due to a relatively small sample size for some meta-analyses, suggestive evidence was set at *P *< 0.10 for the moderator analysis. Strength criteria for the standardized mean difference effect sizes were set as follows: 0.20 (small), 0.50 (medium), 0.80 (large).^44^

## Results

### Systematic review

**Figure 1:**
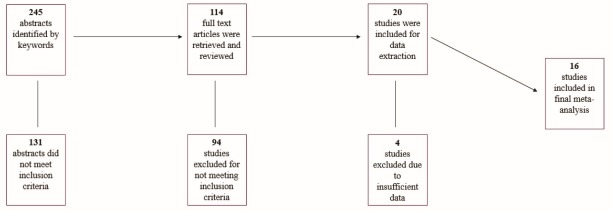
Schematic depiction of the systematic review‏.

Figure 1 displays the results of the systematic review procedures. A total of 245 studies were first identified by keywords. After a complete review of all abstracts, the full-text of 114 articles were retrieved. After review of all full-text articles, 20 studies met inclusion criteria with 16 meeting all final criteria. Table 1 describe these studies in terms of their characteristics.^45-60^ A total of 77 effect sizes were computed from all studies with specific HRQOL measures ranging from 6 (ERF and PRF) to 12 (PF) effect sizes. Table 2 contains results from the PEDro methodological quality analysis. Of the 16 studies included in the analyses, the mean PEDro score of 4.9 (SD = 1.0).

### Meta-analyses

**Figure 2:**
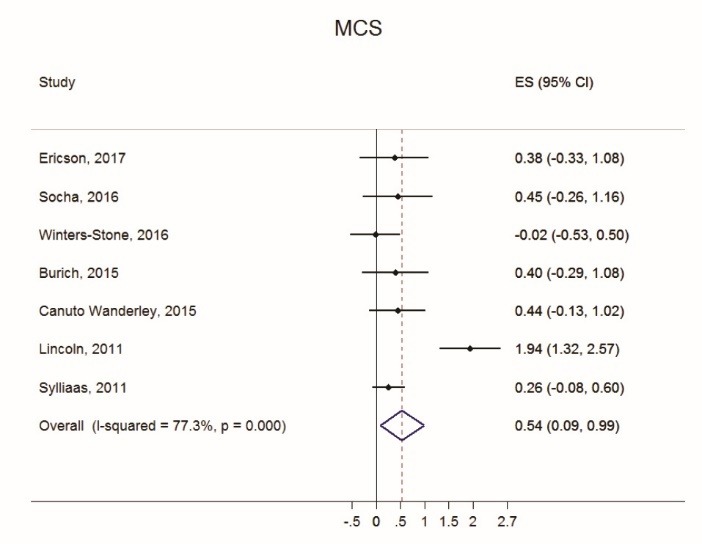
Forest plot of Mental Component Score (MCS) effect sizes and random effects (RE) pooled estimate.

Figures 2 thru 5 display both study-level and pooled mean effect sizes across the ten HRQOL measures. RT showed a positive and significant effect on three of the five mental HRQOL measures, including MCS (ES = 0.54, 95% CI: 0.09-0.99), MH (ES = 0.64, 95% CI: 0.30-0.99), and VT (ES = 0.39, 95% CI: 0.15-0.64). Similarly, RT showed a positive and significant effect on four of the five physical HRQOL measures, including PCS (ES = 0.50, 95% CI: 0.07-0.94), BP (ES = 0.81, 95% CI: 0.26-1.35), GH (ES = 0.57, 95% CI: 0.19-0.94), and PF (ES = 0.40, 95% CI: 0.10-0.71). Of the ten meta-analyses, RT did not appear to significantly effect ERF, SF, and PRF.

Table 3 provides evidence for the heterogeneity of effect sizes across the ten meta-analyses. All *Q* statistics were significant (*P *< 0.01), indicating heterogeneity in effect sizes. Additionally, *I*^2^ values were large for all ten meta-analyses, with the smallest *I*^2^showing approximately 63% variance (inconsistency) in effect sizes due to factors other than sampling error (chance).

### Moderator analyses

Table 4 displays results of the random effects moderator analysis on the mental HRQOL measures. Only a few factors showed moderating effects on the RT and mental HRQOL relationship. The study gender significantly changed effect size measures for MH and SF, with interventions containing both genders showing greater RT effect on MH (ES = 0.85, *P* = 0.026) and interventions containing females only showing greater RT effect on SF (ES = 0.91, *P *< 0.001). Also, noteworthy, intervention length showed a significant and positive relationship with RT effect on ERF (slope = 0.07, *P *< 0.001). Table 5 contains the similar moderator analysis results for the physical HRQOL measures. Intervention multiplicity status significantly changed effect size measures for BP and GH, with RT plus other components showing significantly greater RT effect on BP (ES =1.46, *P*=0.008) and GH (*ES*=1.10, *P*<0.001). Additionally, age categorized into three different groups significantly changed effect size measures for PCS and GH, with those in the 65+ years group seeing lower RT effect on PCS (ES=-0.03, *P*=0.001) and GH (ES=.08, *P*=0.029).

### Sensitivity analyses

Table 6 contains results from the three-step sensitivity analysis. Only two meta-analyses showed signs of funnel plot asymmetry. Although two effect sizes were required to balance the MH meta-analysis, its pooled mean effect size was still significant after imputation (MH: ES=0.48, CI: 0.16-0.80). Conversely, two effect sizes were required to balance the GH meta-analysis, however, its pooled mean effect size was no longer significant after imputation (GH: ES=0.34, CI: -0.03-0.71). Finally, results from the leave-one-out analyses were less consistent. Specifically, seven (MCS, MH, VT, PCS, BP, GH, and PF) of the ten meta-analyses had effects that remained significant regardless of which single study was removed from the pooled mean estimate. This implies that no single study influenced the significance of the effect that RT had on those HRQOL measures. The remaining three meta-analyses (ERF, SF, and PRF) each showed non-significant effects across each study removed with exception of one single study. That is, for each of these three meta-analyses, a single study removed brought the pooled mean estimate to a significant level. Specifically, if Tomas-Carus (2016) is left out of the meta-analyses, RT shows a significant effect on both ERF (ES=0.56, CI: 0.06-1.06) and SF (ES=0.37, CI: 0.06-0.69). Similarly, if Teixeira (2010) is left out of the meta-analysis, the effect that RT has on PRF (ES=0.30, CI: 0.05-0.54) becomes significant. Therefore, considering these sensitivity analysis results, RT intervention is likely to improve all ten measures of HRQOL.

**Figure 3:**
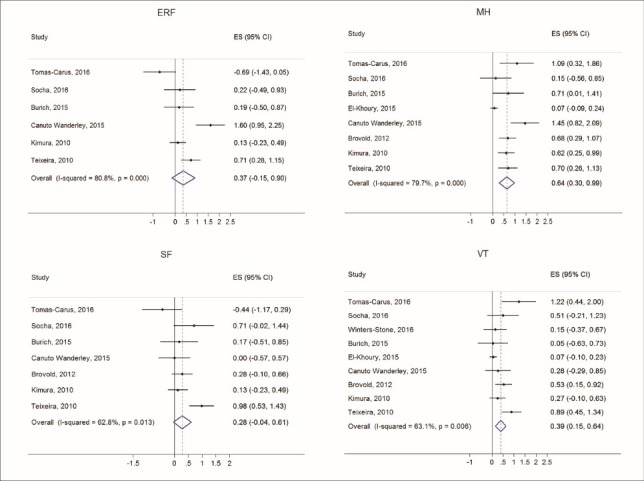
Forest plots of effect sizes and random effects (RE) pooled estimates across mental health dimensions of mental health (MH), emotional role function (ERF), social function (SF), and vitality (VT).

**Figure 4:**
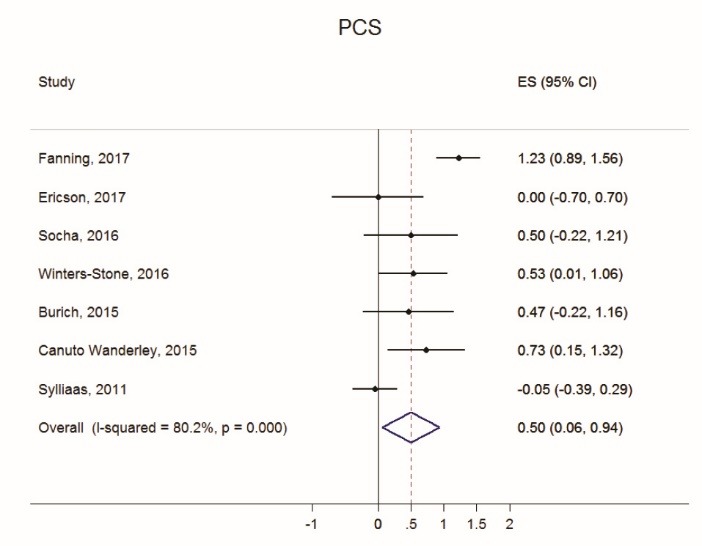
Forest plot of Physical Component Score (PCS) effect sizes and random effects (RE) pooled estimate.

**Figure 5:**
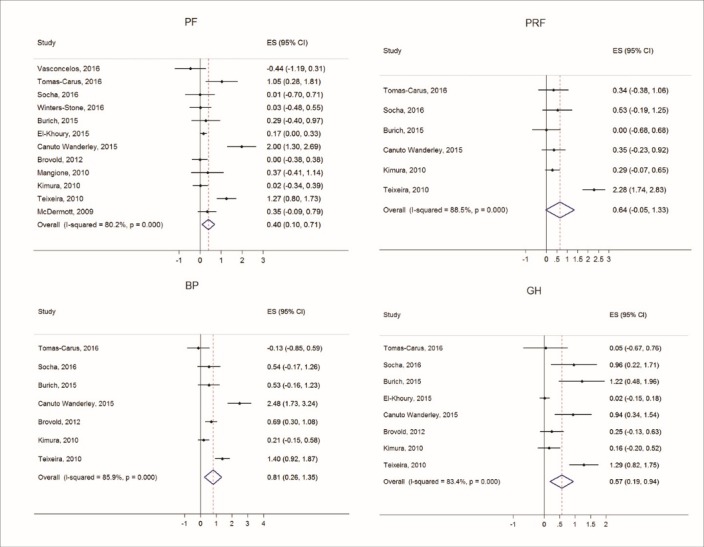
Forest plots of effect sizes and random effects (RE) pooled estimates across physical health dimensions of physical functioning (PF), bodily pain (BP), physical role function (PRF), and general health (GH).

## Discussion

The purpose of this study was to use systematic review and meta-analytic techniques to examine the effect of RT on measures of HRQOL in older adults. Additionally, this research sought to use only HRQOL measures assessed by the gold-standard SF-36/12 assessment which consisted of MCS, ERF, MH, VT, SF, PCS, BP, GH, PF, and PRF. Results from this study support RT intervention as an effective means for improving HRQOL in older adults. These results, however, are not without caveats and, therefore, should be discussed. For instance, three of the ten meta-analyses (ERF, SF and PRF) did not significantly support RT as an efficacious means for increasing HRQOL, at the initial stages of analysis. However, results from the sensitivity analysis revealed a single study was influencing the non-significant pooled mean effects. Specifically, if Tomas-Carus (2016) is removed from the meta-analyses, it is seen that RT has a significant effect on both ERF and SF. This inconsistency is clarified after further inspection into the Tomas-Carus (2016) data. Specifically, Tomas-Carus (2016) reported an unusually low pretest ERF mean value for the control group of 79.5, whereas the treatment group pretest ERF mean was 93.8. Posttest ERF means for control and treatment groups were 92.3 and 92.5, respectively. Conversely, Tomas-Carus (2016) reported an unusually high pretest SF mean value for the treatment group of 89.1, whereas the control group pretest mean was 82.7 for SF. Posttest SF means for treatment and control groups were 82.0 and 82.7, respectively. When examining the Tomas-Carus (2016) data this way, it becomes clearer that a case of statistical regression toward the mean possibly influenced these study findings.^61^ Furthermore, the control group in Tomas-Carus (2016) was a true control group in that they were only instructed to behave in their usual manner. With this in mind, it is unlikely to see a control group experience this amount of ERF improvement and it is more likely that some subjects were measured as having abnormally lower ERF than typical. It is also likely, that this same reasoning (i.e., statistical regression) explains why the treatment group in Tomas-Carus (2016) appeared to suffer such a drop in SF from pretest to posttest.

The third meta-analysis that indicated an initial non-significant effect was concerning PRF. Specifically, if Teixeira (2010) is removed from the meta-analyses, it is seen that RT has a significant effect on PRF. At first glance, the pooled mean effect becoming significant after removing the Teixeira (2010) seems counterintuitive (see Figure 5). It would appear that Teixeira (2010) included in the PRF meta-analysis would, if anything, skew the pooled mean estimate in the positive (greater effect) direction. However, when considering random effects models, the standard error of the pooled mean effect size is a function of not only the inverse variance weights but additionally the variance component of tau-squared (*τ*^2^).^62^ Tau-squared is a measure of between-study variance and adding its component to the inverse variance weights of the standard error computation is the driving mechanism behind random effects models.^63,64^ Therefore, the value of tau-squared was considerably large with Teixeira (2010) in the PRF meta-analysis. More specifically, the Teixeira (2010) effect size contributing to the PRF meta-analysis was 2.28 (CI: 1.74-2.83), which yielded a tau-squared of 0.65 (see Table 3). With such a large tau-squared, the standard error of the pooled mean effect size thus produced an unusually large confidence interval. Hence, the PRF meta-analysis lacked power to find a RT effect. And so, the RT effect size of 0.30 (CI: 0.05-0.54), with Teixeira (2010) omitted, may be a more suitable reported effect on PRF, with the Teixeira (2010) effect size likely belonging to a different population.

With the above caveats explained, it can then be concluded that RT has a robust effect on HRQOL in older adults. This conclusion is supported by findings from similar meta-analyses. A recent meta-analysis examined the effect of RT on HRQOL among participants with chronic heart failure (CHF).^65^ This meta-analysis included studies that used a different HRQOL assessment (Minnesota Living with Heart Failure Questionnaire) with a lower bound mean age of 48 years. The findings from this meta-analysis supported RT as a strong positive factor in increasing HRQOL. Another meta-analysis examined the effect of RT on HRQOL among participants with chronic kidney disease (CKD).^66^ In this research, measures of HRQOL were extracted from studies that used the PF and PCS of the SF-36 assessment with a lower bound mean age of 43 years. Results from this meta-analysis also supported RT as an effective intervention in improving HRQOL in participants. A final study worth noting is a meta-analysis that examined the effect of RT on HRQOL in cancer patients.^67^ This meta-analysis included studies that used two different disease-specific HRQOL assessments, the Functional Assessment of Cancer Therapy (FACT) self-report questionnaire and the Cancer Rehabilitation Evaluation System Short Form (CARES-SF). Only six studies were included in this meta-analysis, with a lower bound mean age of 49 years. However, a small RT effect (ES=-0.17 in favor of intervention) on HRQOL was still seen. Given the results from these supporting studies and results from the current meta-analyses, RT clearly is an effective intervention for increasing HRQOL in older adults.

The major strength of this study was its use of the SF-36/12 assessment as inclusion criteria during the systematic review. This inclusion criteria gave strength to this research for two reasons. One, the SF-36/12 assessment, as previously mentioned, is a gold standard HRQOL assessment in physical activity research, providing both valid and reliable measures.^28,30^ Two, the SF-36/12 assessment has a unique attribute in that it allows for ten different HRQOL scores.^29^ This attribute of the SF-36/12 assessment permits a greater and more valid coverage of the various health-related dimensions that ultimately affect the quality of life of older adults.

This study does have limitations worth noting. First, this study is possibly limited due to the phenomenon of publication bias.^68^ Publication bias exists in a meta-analysis if studies with negative (null) findings have been systematically omitted from the data extraction process. However, this phenomenon is more likely to occur in industries such as pharmaceutical manufacturing, where organizations have a stake in the research results.^69^ In physical activity research, a null finding is more likely considered a valuable addition to the literature. For example, of the 77 effects extracted from the 16 studies in this research, 49 were non-significant – which arguably is evidence against publication bias. Additionally, bias was addressed in this research during the sensitivity analysis, where little bias was found. Second, this study is possibly limited due to search bias. That is, a bias introduced using a limited search strategy. Although this limitation is important to consider, this study took measures to prevent search bias. Specifically, the systematic review procedures included a search of the PubMed database as well as included a large set of keyword terms to ensure a sensitive search. Third, this study is possibly limited due to selection bias, which is related to bias in the way flagged abstracts and articles were included into the meta-analysis. This limitation is important to consider. However, this study utilized two independent researchers on all stages of the systematic review and data extraction procedures, to limit this potential bias. Finally, the use of a single database (i.e., PubMed) may have limited this research and decreased the quality of the search strategy by systematically missing relevant research articles. However, PubMed, a web-based portal of MEDLINE developed by the United States Department of Health and Human Services, has been shown to be more effective with comprehensive medical-related reviews than other similar databases.^70^

## Conclusion

The meta-analytic evidence presented in this research clearly supports RT as an effective means for improving HRQOL in older adults. The array of specific HRQOL dimensions that RT may improve span both mental (MCS, ERF, MH, VT, and SF) and physical (PCS, BP, GH, PRF, and PF) HRQOL domains. RT may, however, be particularly effective at improving MH and BP in older adults. RT should be a priority intervention for improving HRQOL in older adults and helping to meet our national HRQOL goals.

## Ethical approval

This study used already published data from journal articles. Therefore, institutional review board approval was not required.

## Competing interests

The authors declare that they have no competing interests.

## Authors’ contributions

PDH and DJB designed, managed, and analyzed this study as well as wrote this paper.

## Funding

No funding was provided for this research.

## Acknowledgments

The authors would like to thank Dillon Barnes for helping with the beginning stages of this research.

**Table 1:** Characteristics of included meta-analysis studies

**First Author**	**Year**	**Measure**	**Form**	**Mean Age (yr)**	**Age Group (y)**	**Gender**	**Population**	**Length (wk)**	**Combined**
Fanning J^45^	2017	PCS	SF-12	66.9	60+	Both	Obese	24	Yes
Ericson H^46^	2017	MCS, PCS	SF-12	67.5	65+	Female	Healthy	24	No
Vasconcelos KS^47^	2016	PF	SF-36	72.0	65+	Female	Sarcopenic obese	10	No
Tomas-Carus P^48^	2016	BP, ERF, GH, MH, PF, PRF, SF, VT	SF-36	59.9	50+	Both	Diabetic	12	Yes
Socha M^49^	2016	BP, ERF, GH, MH, PF, PRF, SF, VT, PCS, MCS	SF-36	62.5	50+	Female	PM	8	No
Winters-Stone KM^50^	2016	MCS, PCS, PF, VT	SF-36	70.6	60+	Both	Healthy	24	No
Burich R^51^	2015	BP, ERF, GH, MH, PF, PRF, SF, VT, PCS, MCS	SF-36	62.7	50+	Both	Healthy	12	Yes
El-Khoury F^52^	2015	GH, MH, PF, VT	SF-36	79.8	65+	Female	Fall risk	104	Yes
Canuto Wanderley FA^53^	2015	BP, ERF, GH, MH, PF, PRF, SF, VT, PCS,MCS	SF-36	68.0	60+	Both	Healthy	32	No
Brovold T^54^	2012	BP, GH, MH, PF, SF, VT	SF-36	79.0	60+	Both	Acute problem	12	Yes
Lincoln AK^55^	2011	MCS	SF-36	67.1	60+	Both	Diabetic	16	No
Sylliaas H^56^	2011	MCS, PCS	SF-12	82.1	65+	Both	Hip surgery	12	No
Mangione KK^57^	2010	PF	SF-36	79.6	65+	Both	Hip surgery	10	No
Kimura K^58^	2010	BP, ERF, GH, MH, PF, PRF, SF, VT	SF-36	73.6	65+	Both	Healthy	12	Yes
Teixeira LE^59^	2010	BP, ERF, GH, MH, PF, PRF, SF, VT	SF-36	63.1	50+	Female	PM/OP	18	No
McDermott MM^60^	2009	PF	SF-36	71.7	60+	Both	PAD	24	No

*Note.* BP is bodily pain. ERF is emotional role functioning. GH is general health. MH is mental health. PF is physical functioning. PRF is physical role functioning. SF is social functioning. VT is vitality. MCS is mental component score. PCS is physical component score. PM is postmenopausal. OP is osteoporosis. PAD is peripheral arterial disease.

**Table 2:** Physiotherapy Evidence Database (PEDro) scores for included meta-analysis studies

**HRQOL**	**N**	**Mean**	**SD**
Overall	16	4.9	1.0
Measure			
BP	7	4.3	1.1
ERF	6	4.2	1.2
GH	8	4.4	1.1
MCS	7	4.9	1.2
MH	8	4.4	1.1
PCS	7	5.1	1.2
PF	12	4.8	1.1
PRF	6	4.2	1.2
SF	7	4.3	1.1
VT	9	4.6	1.1

*Note.* N indicates number of studies. SD is standard deviation. PEDro scores can typically range from 0 to 10 but can only range from 0 to 7 in this research.

**Table 3:** Summary results and variance components across SF-36/12 component scores and dimensions

**HRQOL measure**	**N**	**ES**	**95% CI**	**τ** ^ 2 ^	**I** ^ 2 ^	**Q**
Mental						
MCS	7	0.54	0.09-0.99	0.28	77.3	26.4
ERF	6	0.37	-0.15-0.90	0.34	80.8	26.1
MH	8	0.64	0.30-0.99	0.18	79.7	34.4
VT	9	0.39	0.15-0.64	0.08	63.1	21.7
SF	7	0.29	-0.04-0.61	0.11	62.8	16.1
Physical						
PCS	7	0.50	0.07-0.94	0.27	80.2	30.3
BP	7	0.81	0.26-1.35	0.45	85.9	42.5
GH	8	0.57	0.19-0.94	0.22	83.4	42.2
PRF	6	0.64	-0.05-1.33	0.65	88.5	43.4
PF	12	0.40	0.10-0.71	0.21	80.3	55.7

*Note. N* represents number of studies. *Q* statistic (with *N*-1 *df*) tests for heterogeneity. All *Q P* values were significant at *P*<0.01. *τ*^2^ represents variance component. *I*^2^ represents percent of heterogeneity.

**Table 4:** Effect size by moderator for the SF-36 and SF-12 MCS and mental health dimensions

**Moderator**	**MCS**	***P***	**MH**	***P***	**SF**	***P***	**ERF**	***P***	**VT**	***P***
Gender^a^		0.761		**0.026**		**<0.001**		0.788		0.866
Female	0.41		0.28		0.91		0.49		0.43	
Both	0.58		0.85		0.11		0.31		0.38	
Health Problem^a^		0.600		0.623		0.710		0.491		0.444
Yes	0.69		0.56		0.36		0.07		0.49	
No	0.42		0.73		0.22		0.52		0.27	
Multiplicity^a^		0.819		0.561		0.091		**0.032**		0.582
RT only	0.40		0.57		0.09		-0.09		0.34	
RT plus	0.56		0.78		0.59		0.85		0.48	
Age Group^a^		0.758		0.205		0.808		0.087		0.051
50+	0.42		0.66		0.40		0.15		0.69	
60+	0.77		1.00		0.16		1.60		0.36	
65+	0.31		0.31		0.13		0.13		0.14	
Length^b^		0.726		0.054		0.818		**<0.001**		0.097
Slope	-0.01		-0.01		-0.01		0.07		-0.01	
Age^b^		0.594		0.254		0.845		0.479		0.062
Slope	-0.02		-0.02		-0.01		0.05		-0.03	
PEDro Score^b^		0.192		0.937		0.712		0.823		0.546
Slope	-0.25		-0.02		0.06		0.06		-0.08	

*Note.* All values under HRQOL measures are ES. All moderator analyses were performed using random effects models with non-pooled variances (tau-squared). ^a^These moderators are treated as categorical with group-specific mean effect sizes reported. ^b^These moderators are treated as continuous with meta-regression coefficients reported. *P* values in bold are significant at *P*<0.05. *P* values underlined are suggestive at *P*<0.10.

**Table 5:** Effect size by moderator for the SF-36 and SF-12 PCS and physical health dimensions

**Moderator**	**PCS**	***P***	**BP**	***P***	**GH**	***P***	**PRF**	***P***	**PF**	***P***
Gender^a^		0.524		0.671		0.652		**0.015**		0.644
Female	0.29		1.00		0.71		1.48		0.30	
Both	0.59		0.73		0.50		0.25		0.47	
Health Problem^a^		0.770		0.719		0.358		0.054		0.582
Yes	0.57		0.68		0.40		1.38		0.34	
No	0.43		0.91		0.77		0.29		0.54	
Multiplicity^a^		0.063		**0.008**		**<0.001**		0.185		0.366
RT only	0.95		0.35		0.22		0.21		0.25	
RT plus	0.32		1.46		1.10		1.07		0.53	
Age Group^a^		**0.001**		0.366		**0.029**		0.884		0.229
50+	0.48		0.61		0.93		0.80		0.68	
60+	0.92		1.53		0.53		0.35		0.52	
65+	-0.03		0.21		0.08		0.29		0.04	
Length^b^		0.345		**<0.001**		0.298		0.802		0.943
Slope	0.02		0.10		-0.01		0.01		0.00	
Age^b^		0.222		0.901		**0.006**		0.708		0.059
Slope	-0.04		0.01		-0.05		-0.03		-0.04	
PEDro Score^b^		0.995		0.680		0.578		0.704		0.429
Slope	0.00		0.12		0.12		0.14		-0.13	

*Note.* All values under HRQOL measures are ES. All moderator analyses were performed using random effects models with non-pooled variances (tau-squared). ^a^These moderators are treated as categorical with group-specific mean effect sizes reported. ^b^These moderators are treated as continuous with meta-regression coefficients reported. *P* values in bold are significant at *P*<0.05. *P* values underlined are suggestive at *P*<0.10.

**Table 6:** Sensitivity analysis of effect sizes across SF-36 and SF-12 component scores and dimensions

**HRQOL measure**	**Asymmetry** ^a^		**Trim-and-fill** ^b^			**Leave-one-out** ^c^			
	***Z***	***P***	**#** ^d^	**ES** ^e^	**95% CI**	**ES**_L_^f^	**95% CI**	**ES**_H_^g^	**95% CI**
Mental									
MCS	0.59	0.557	0	0.54	0.09-0.99	0.28	0.06-0.50	0.64	0.13-1.15
ERF	-0.37	0.712	0	0.37	-0.15-0.90	0.16	-0.25-0.57	0.56^h^	0.06-1.06
MH	2.16	0.031	2	0.48	0.16-0.80	0.53	0.22-.84	0.74	0.50-0.98
VT	1.43	0.153	0	0.39	0.15-0.64	0.30	0.09-0.52	0.47	0.23-0.70
SF	-0.55	0.579	1	0.37	0.04-0.71	0.16	-0.06-0.38	0.37^h^	0.06-0.69
Physical									
PCS	-0.42	0.673	0	0.50	0.07-0.94	0.32	0.03-0.61	0.63	0.25-1.02
BP	0.55	0.582	0	0.81	0.26-1.35	0.57	0.15-0.99	0.95	0.37-1.53
GH	1.98	0.048	2	0.34	-0.03-0.71	0.42	0.10-0.74	0.67	0.27-1.07
PRF	-0.29	0.770	2	0.89	0.31-1.47	0.30^i^	0.05-0.54	0.76	-0.02-1.55
PF	0.83	0.408	0	0.40	0.10-.71	0.27	0.03-0.52	0.46	0.15-0.78

*Note.* ^a^Egger’s regression models for testing funnel plot asymmetry. ^b^Trim-and-fill method for estimating the number of effect sizes required to show a symmetric funnel plot. ^c^Leave-one-out analysis re-estimating the effect sizes once for each study deleted. ^d^Number of effect sizes needed to balance the funnel plot. ^e^Estimated mean effect size with imputed study effect sizes needed to balance funnel plot. ^f^Lowest *ES* seen from leave-one-out analysis. ^g^Highest effect size seen from leave-one-out analysis. ^h^If Tomas-Carus (2016) is left out of the meta-analysis, both ERF (ES=0.56) and SF (ES=0.37) effects become significant. ^i^If Teixeira (2010) is left out of the meta-analysis, PRF (ES=0.30) effect becomes significant.
